# Cell composition at the vitreomacular interface in traumatic macular holes

**DOI:** 10.1007/s00417-021-05470-z

**Published:** 2021-11-03

**Authors:** Stefanie R. Guenther, Ricarda G. Schumann, Yulia Zaytseva, Felix Hagenau, Armin Wolf, Siegfried G. Priglinger, Denise Vogt

**Affiliations:** 1 Department of Ophthalmology, Ludwig-Maximilians-University, Vitreoretinal Pathology Unit, Mathildenstrasse 8, 80336 Munich, Germany; 2 Munich Eye Center Brienner Hof, Munich, Germany; 3 Department of Ophthalmology, University of Ulm, Ulm, Germany

**Keywords:** Traumatic macular hole, Internal limiting membrane, Membrane peeling, Vitreomacular interface, Immunocytochemistry, Transmission electron microscopy

## Abstract

**Purpose:**

To describe characteristics of the vitreomacular interface (VMI) in traumatic macular holes (TMH) compared to idiopathic macular holes (IMH) using immunofluorescence and electron microscopy, and to correlate with clinical data.

**Methods:**

For immunocytochemical and ultrastructural analyses, premacular tissue with internal limiting membrane (ILM) and epiretinal membrane (ERM) was harvested during vitrectomy from 5 eyes with TMH and 5 eyes with IMH. All specimens were processed as flat mounts for phase-contrast microscopy, interference and fluorescence microscopy, and transmission electron microscopy (TEM). Primary antibodies were used against microglial and macroglial cells. Clinical data was retrospectively evaluated.

**Results:**

Surgically excised premacular tissue of eyes with TMH showed a less pronounced positive immunoreactivity for anti-glutamine synthetase, anti-vimentin and anti-IBA1 compared to eyes with IMH. Cell nuclei staining of the flat-mounted specimens as well as TEM presented a lower cell count in eyes with TMH compared to IMH. All detected cells were found on the vitreal side of the ILM. No collagen fibrils were seen in specimens of TMH. According to patients’ age, intraoperative data as well as spectral-domain optical coherence tomography (SD-OCT) analysis revealed an attached posterior vitreous in the majority of TMH cases (60%), whereas all eyes with IMH presented posterior vitreous detachment.

**Conclusion:**

The vitreomacular interface in TMH and IMH shows significant differences. In TMH, glial cells are a rare finding on the vitreal side of the ILM.

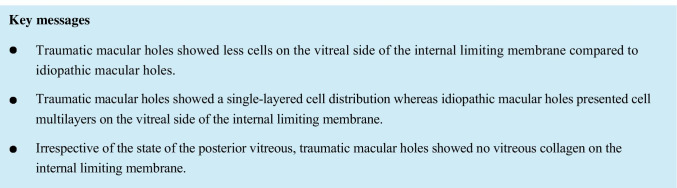

## Introduction

The traumatic macular hole (TMH) is a rare full-thickness retinal tissue defect of the fovea with an interruption of all neural retinal layers leading to severe vision loss and includes 5–8.2% of all various types of macular holes [1–3]. Traumatic macular hole can develop either immediately after an injury or during the weeks following the trauma with an incidence of 0.15% after open eye injury and in 1.4% after blunt ocular trauma [2, 4–6] [7, 8]. The prevalence of TMH is statistically higher in men than in women (86.3% vs. 27.7%) and the affected patients are in general of a younger age with a peak in children and adolescents [1, 9–11].

Unlike in idiopathic macular holes (IMH), spontaneous closure is common in TMH. In the literature, the spontaneous closure rates vary between 10 and 50% [7, 12–20]. Functional prognosis is often limited due to accompanying trauma-induced retinal pathologies, respectively. Possible associated retinal pathologies are retinal or vitreous haemorrhage, choroidal rupture, damage of the retinal pigment epithelium (RPE), commotio retinae, subretinal choroidal neovascularisation and fibrosis [5].

The pathogenesis of TMH is still under discussion. The most common theory implies that anteroposterior compression and equatorial expansion of the globe appear to result in retinal stress, which may result in a full-thickness macular defect without loss of foveal retinal tissue [6, 8, 9]. Additionally, vitreoretinal traction caused by an ocular trauma contributes to macular hole development.

However, there is less information about cellular components at the vitreomacular interface in these traumatic types of macular holes. To our knowledge, there is only one case series with one analysed TMH specimen presenting positive immunostaining of glial fibrillary acidic protein (GFAP) and neuron-specific enolase (NSE) [21]. Immunostaining of alpha smooth muscle actin (SMA) and CD68 was not found by Kanavi et al. [21]. To date, the ultrastructure of premacular tissue in TMH has only been investigated by Kumar and his colleagues, in which they described the presence of ILM in 3 of 3 specimens as well as native vitreous collagen (NVC) in 1 of 3 specimens [22]. But neither cells nor epiretinal membranes (ERM) were seen in transmission electron microscopy (TEM) [22].

Previous studies showed an attached posterior vitreous body in the majority of eyes with TMH using SD-OCT and clinical examination [2, 8, 10, 23]. In contrast, posterior vitreous detachment (PVD) with its anteroposterior and tangential vitreomacular traction is identified as the major cause in pathogenesis of IMH [24–26]. Consistent with these findings, hyalocytes, glial cells and myofibroblast-like cells are demonstrated as the main cell types in eyes with IMH using immunocytochemistry and transmission electron microscopy (TEM) [27–29].

The aim of our study was to firstly identify vitreomacular interface changes in surgically excised premacular tissue of eyes with TMH by immunocytochemistry and TEM and then compare the results with the findings in IMH. For clinical correlation, demographic data and SD-OCT findings of both entities were retrospectively analysed.

## Materials and methods

In this clinicopathological study, surgically excised premacular tissue of internal limiting membrane (ILM) and epiretinal membrane (ERM) from eyes with TMH (5 eyes) and eyes with IMH (5 eyes) was consecutively obtained by three different surgeons during vitrectomy at the Ludwig-Maximilians-University, Department of Ophthalmology, between April 2016 and January 2020. All 10 specimens were processed as flat mounts for phase contrast, interference and immunocytochemistry. All 10 specimens were also processed for ultrastructural cell analysis using transmission electron microscopy (TEM). Based on the size of the macular hole diameter using preoperative spectral-domain optical coherence tomography (SD-OCT), control specimens of IMH were selected comparable to specimens of TMH. Clinical data as well as SD-OCT scans using Heidelberg Spectralis OCT (Heidelberg Engineering, Heidelberg, Germany) were retrospectively analysed.

The Institutional Review Board and the Ethics Committee of the Ludwig-Maximilians-University, Munich, approved the surgical removal as well as the histopathologic preparation and analysis of the patients’ specimens (No. 471–14). Informed consent was obtained from each patient. The study was conducted according to the tenets of the Declaration of Helsinki.

The included patients presented with a reduced visual acuity and full-thickness macular holes on biomicroscopy and showed a full-thickness macular defect on SD-OCT. All patients with TMH reported an ocular trauma shortly before the reduction of visual acuity. Besides, typical SD-OCT characteristics for TMH, such as eccentric, ellipsoid-shaped full-thickness macular defects, a large basal macular hole diameter, little to no intraretinal fluid or additional defects like rupture of retinal pigment epithelium (RPE), were found [5, 10, 23]. The SD-OCT scans of the included patients with IMH showed intraretinal fluid, increased retinal thickness on the edges of the macular hole in comparison to neighbouring retinal tissue and almost round-shaped full-thickness macular defects [10, 25]. The included IMH showed a diameter of more than 250 µm. Patients with IMH, who referred to an ocular trauma in the past, were excluded.

Patients’ charts were reviewed for age, sex, preoperative and postoperative best corrected visual acuity (BCVA), period of time between diagnosis and surgery, and the follow-up period (Table 1). Using SD-OCT, we analysed the macular hole diameter at the narrowest point of the hole with the caliper function of the SD-OCT device drawing a line roughly parallel to the RPE, state of posterior hyaloid, intraretinal fluid, vitreomacular traction (VMT), ERM and possible defects of the RPE at baseline. For the state of posterior hyaloid, intraoperative assessment of the surgeon was considered as well as the posterior vitreous detachment seen in the central foveal scan by SD-OCT. At the last follow-up, biomicroscopy and SD-OCT were used to study whether the full-thickness macular hole was closed after the performed surgery. Patient number 10 was lost to follow-up, and therefore, the postoperative state of their IMH remains unknown.

**Table 1:** Clinical and follow-up data and spectral-domain optical coherence tomography characteristics at baseline

Patient data					SD-OCT characteristics at baseline					BCVA (LogMAR)			Follow-up	
No	Age (years)	Sex	Diagnosis	FTMH diameter	Posterior vitreous detachment (PVD)	Intraretinal fluid on FTMH edges	Vitreomacular traction	Epiretinal membrane	RPE defects	Preoperative	Postoperative	Time between diagnosis and surgery (months)	Follow-up period (months)	FTMH closure after first vitrectomy
1	20	M	TMH	283	No PVD	Yes	No	No	No	0.7	1.0	1	2	Yes
2	17	F	TMH	527	No PVD	Yes	No	No	Yes	0.7	0.6	2	3	No**
3	16	M	TMH	513	No PVD	No	No	No	No	0.6	0.2	1	17	Yes
4	74	M	TMH	320	Complete PVD	Yes	No	Yes	No	1.0	1.2	144	10	No***
5	68	M	TMH	601	Incomplete PVD	Yes	No	No	No	1.2	0.5	4	1	Yes
6	67	F	IMH	389	Complete PVD	Yes	No	Yes (peripheral)	No	0.7	0.3	6	2	Yes
7	71	M	IMH	368	Incomplete PVD	Yes	VMA	Yes	No	0.8	0.4	1	6	Yes
8	76	F	IMH	383	Incomplete PVD	Yes	No	Yes (peripheral)	No	1.0	0.5	1	7	Yes
9	80	M	IMH	341	Incomplete PVD	Yes	VMA	Yes (peripheral)	No	0.8	0.6	4	8	Yes
10	60	F	IMH	417	Complete PVD	Yes	No	Yes	No	1.0	2.0*	1	0	Unknown (lost to follow-up)

*SD-OCT*, spectral domain optical coherence tomography; *M*, male; *F*, female; *TMH*, traumatic macular hole; *IMH*, idiopathic macular hole; *FTMH*, full-thickness macular hole; *PVD*, posterior vitreous detachment; *VMA*, vitreomacular adhesion, *RPE*, retinal pigment epithelium, *BCVA*, best corrected visual acuity

^*^BCVA testing 2 days after surgery with 90% of the vitreous cavity filled with C2F6. **TMH closure with second surgery 3 weeks after initial vitrectomy. ***The patient refused a second vitrectomy

The performed surgical technique was a standard 23 gauge pars plana vitrectomy with sequential ILM peeling using end-gripping forceps. For membrane peeling, a vital dye of 0.25 mg/mL solution of Brilliant Blue (Brilliant Peel; Fluoron GmbH, Neu-Ulm, Germany) was used. At the end of surgery, the vitreous cavity was filled with a tamponade of either air, 20% diluted SF6 or 16% diluted C2F6. For transfer, the tissue was kept in a balanced salt solution (BSS) (Bausch and Lomb, Germany).

### Immunocytochemistry

After fixation, specimens were flattened and unfolded showing the maximum surface area using a stereomicroscope (MS 5; Leica, Wetzlar, Germany). Antifading mounting medium 4′,6-diamidino-2-phenylindole (DAPI; AKS-38448; Dianova, Hamburg, Germany) was used to stain the cell nuclei. Primary antibodies were used to identify retinal microglial cells with anti-ionised calcium-binding adaptor molecule 1 (anti-IBA 1) (Rabbit, 019–19,741, Wako Chemicals GmbH, Neuss, Germany) and macroglial cells with anti-glutamine synthetase (anti-GS) (Mouse, GTX84426, GeneTex, Inc., CA 92,606, USA) and anti-vimentin (anti-VIM) (Goat, V 4630, Sigma-Aldrich Chemie GmbH, Taufkirchen, Germany) as listed in Table 2. According to the manufacturer’s instructions, specimens were labelled with a combination of three primary antibodies. Specimens were then incubated with 0.1% pepsine in 0.1 M phosphate-buffered saline (PBS) and normal donkey serum (1:20) in PBS, 0.5% bovine serum albumin (BSA), 0.1% Triton X-100 and 0.1% Na azide. A second antibody, either donkey anti-rabbit Cy2, donkey anti-mouse Cy3, or donkey anti-goat Cy5 (Dianova, Hamburg, Germany), was added after incubation together with the primary antibodies overnight at room temperature. The dilution for the primary antibody was 1:50 and for the secondary antibody 1:100. Furthermore, the flat mounts were analysed for cell count and ILM area.

**Table 2:** Immunocytochemical and ultrastructural characteristics of premacular tissue of 5 eyes with traumatic macular holes and 5 eyes with idiopathic macular holes

	Specimen characteristics		Immunocytochemistry				Transmission electron microscopy			
No	Cell count	Area (mm^2^)	Anti-IBA 1	Anti-glutamin synthetase (anti-GS)	Anti-vimentin (anti-VIM)	Colocalisation	ILM (yes/no)	Cell distribution	Vitreous collagen	
			Microglial cell marker	Mueller cell marker	Macroglial cell marker					
1	5	3.739	-	-	-	-	Yes	No cells	No collagen	
2	14	1.218	+	-	-	Anti-IBA 1 + anti-VIM	Yes	Single layer	No collagen	
3	62	6.594	+	+	+	Anti-GS + anti-IBA-1 + anti-VIM; Anti-IBA-1 + anti-VIM	Yes	Single layer	No collagen	
4	0	1.216	-	-	-	-	Yes	No cells	No collagen	
5	115	4.420	+	+	-	Anti-GS + Anti-IBA 1	Yes	Single layer	No collagen	
6	39	0.310	+	+	+	-	Yes	No cells	No collagen	
7	43	1.687	+	+	+	Anti-GS + anti-VIM	Yes	Multilayer, cell clusters	No collagen	
8	454	0.975	+	+	+	-	Yes	Multilayer	Native vitreous collagen	
9	34	2.238	+	+	+	Anti-GS + anti-VIM	Yes	Multilayer, cell clusters	No collagen	
10	252	1.597	+	+	-	Anti-GS + anti-IBA 1	Yes	Multilayer, cell clusters	Native vitreous collagen	

Nos. 1–5 = eyes with traumatic macular holes (TMH); Nos. 6–10 = eyes with idiopathic macular holes (IMH); - = negativ immunostaining; +  = positive immunostaining

Preparing negative controls, all specimens were dissected into pieces and the primary antibody was substituted with both diluent and isotype controls (IgG2a monoclonal mouse antibodies, X0934, DAKO, Hamburg, Germany; M5409, Sigma-Aldrich, Taufkirchen, Germany). All other procedures were identical to the procedures described above.

### Transmission electron microscopy

For ultrastructural analysis, dehydration in graded concentrations of ethanol and embedding in Epon 812 were performed directly after post fixation with 2% osmium tetroxide (Dalton’s fixative). For staining of the semi-thin sections of 400 nm, an aqueous mixture of 1% toluidine blue and 2% sodium borax was used. Ultrathin series sections of 60 nm followed and were contrasted with uranyl acetate and lead citrate. Using a Zeiss light microscope and a Zeiss EM 9 S-2 electron microscope (Zeiss, Jena, Germany), 5 grids (each with 8–10 ultrathin sections) per specimen were analysed.

## Results

**Fig. 1:**
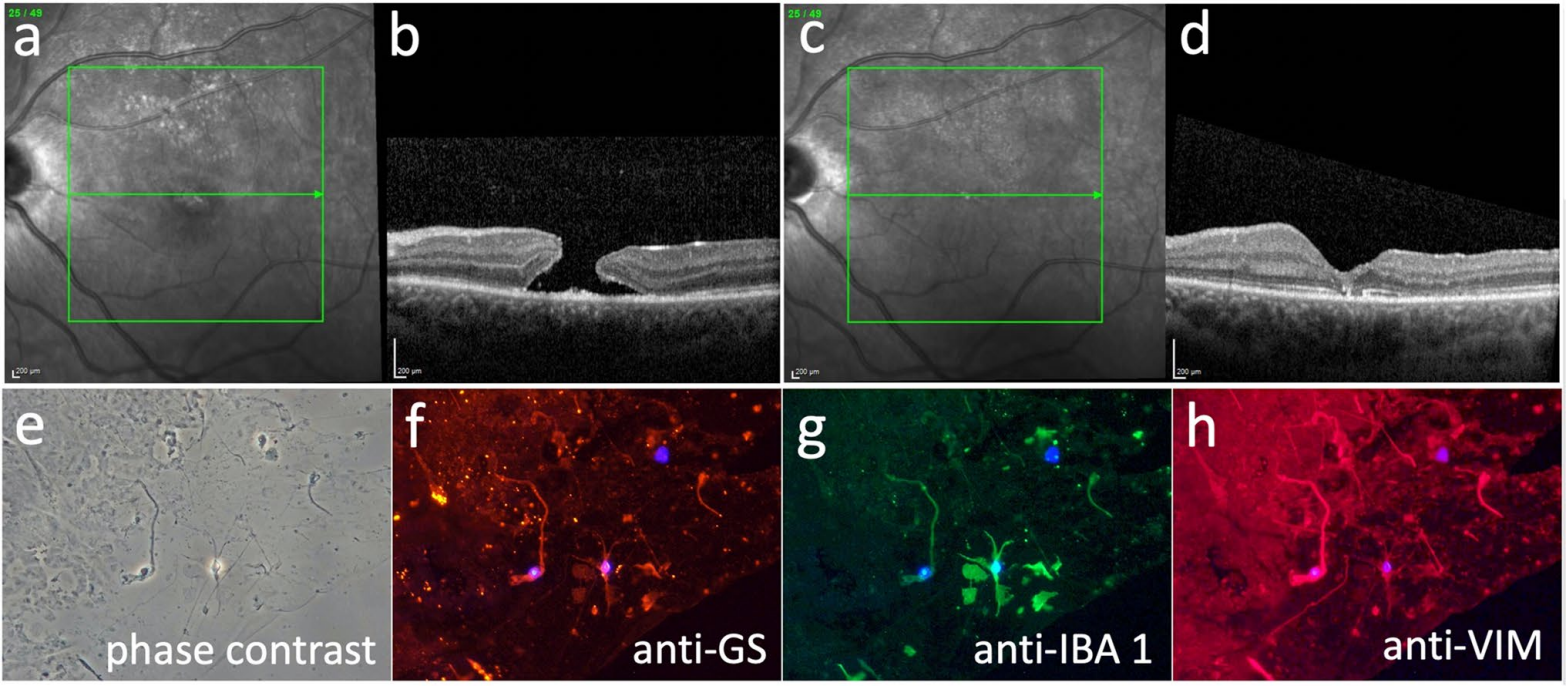
Patient No. 3 with traumatic macular hole (TMH) in the left eye. Preoperative spectral-domain optical coherence tomography (SD-OCT) with infrared image (**a**) and B-scan (**b**) showing a TMH. Infrared image (**c**) and SD-OCT B-scan (**d**) 17 months after pars plana vitrectomy with peeling of premacular tissue including internal limiting membrane (ILM), the former TMH is closed and a foveal depression is seen. **e** Phase contrast of patient no. 3. **f**–**h** Immunocytochemical staining in fluorescence microscopy merged with cell nuclei staining of 4′,6-diamidino-2-phenylindole (DAPI, blue) in flat-mounted surgically excised premacular tissue containing inner limiting membrane (ILM) and epiretinal membrane (ERM) of patient no. 3. A positive immunoreactivity for all three analysed micro- and macroglia markers anti-glutamine synthetase (anti-GS), anti-IBA 1 and anti-vimenin (anti-VIM) can be seen. Original magnifications: **e**–**h** × 400

**Fig. 2:**
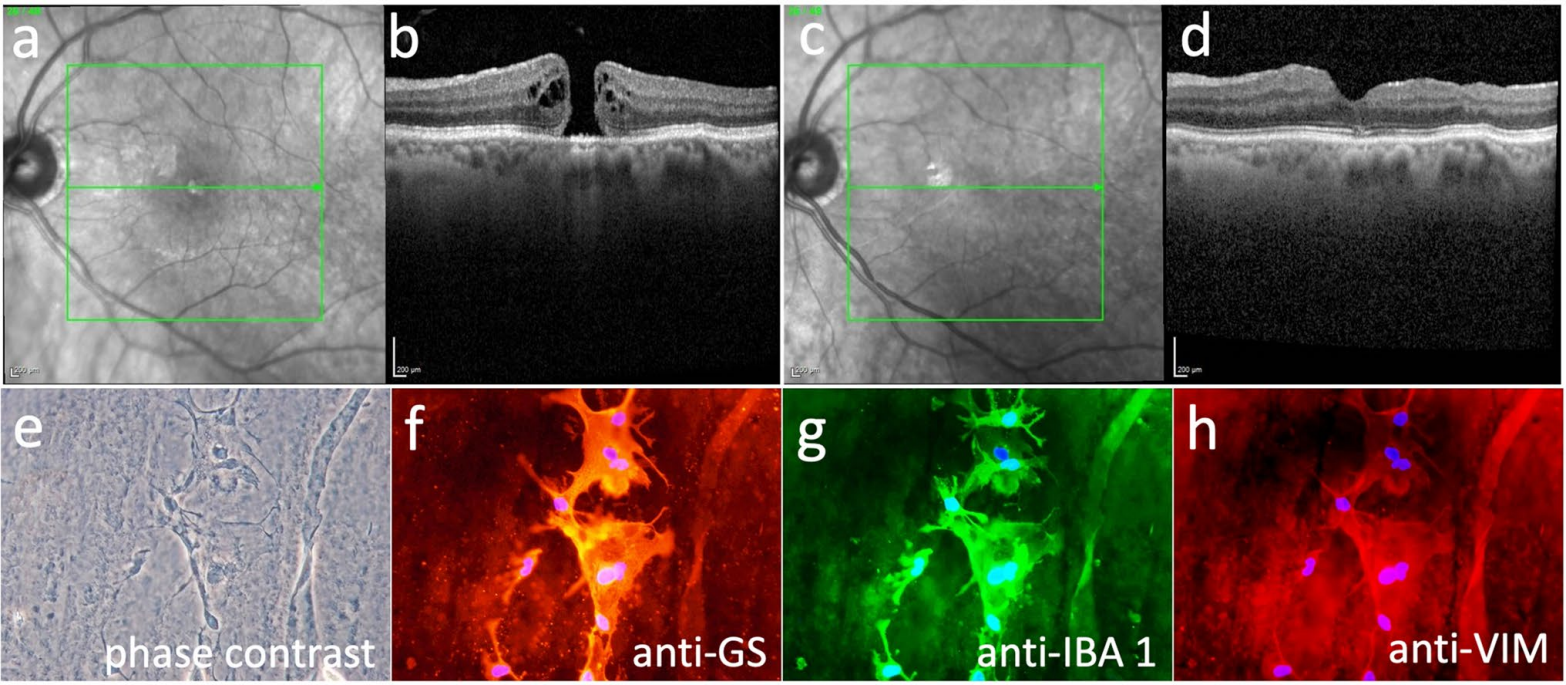
Patient No. 9 with idiopathic macular hole (IMH). Preoperative spectral-domain optical coherence tomography (SD-OCT) with infrared image (**a**) and B-scan (**b**) showing an IMH with intraretinal fluid on the macular hole margins. Infrared image (**c**) and SD-OCT B-scan (**d**) 7 months after pars plana vitrectomy with peeling of premacular tissue including internal limiting membrane (ILM), the former IMH is closed, intraretinal fluid is absent and a foveal depression is seen. **e** Phase contrast of patient no. 9. **f**–**h** Immunocytochemical staining in fluorescence microscopy merged with cell nuclei staining of 4′,6-diamidino-2-phenylindole (DAPI, blue) in flat-mounted surgically excised premacular tissue containing inner limiting membrane (ILM) and epiretinal membrane (ERM) of patient no. 9. A positive immunoreactivity for all three analysed micro- and macroglia markers anti-glutamine synthetase (anti-GS), anti-IBA 1 and anti-vimenin (anti-VIM) can be seen. Original magnifications: **e**–**h** × 400

In this interventional laboratory investigation, we included surgically excised premacular tissue of ILM and ERM from 4 women (1 eye with TMH and 3 eyes with IMH) and 6 men (4 eyes with TMH and 2 eyes with IMH), corresponding to 5 right and 5 left eyes. The clinical data as well as the analysed SD-OCT characteristics are given in Table 1. Exemplary pre- and postoperative SD-OCT scans of a patient with TMH (Fig. 1a–d) and a patient with IMH (Fig. 2a–d) are shown in Figs. 1 and 2.

The patients’ mean age at the time of surgery was 39.0 ± 29.3 SD years (median 20 years, ranged from 16 to 74 years) in TMH and 70.8 ± 7.8 SD years (median 71 years, ranged from 60 to 80 years) in IMH. The mean macular hole diameter was 448.8 ± 139.2 SD µm in TMH and 379.6 ± 27.9 SD µm in IMH. The vitreous was attached to the posterior pole in 3 of 5 eyes (60%) with TMH. One eye with TMH showed a complete PVD (20%) and one eye showed an incomplete PVD (20%). All eyes with IMH showed a PVD, of which a complete PVD was seen in 2 eyes (40%) and an incomplete PVD in 3 eyes (60%). The mean period of the time between diagnosis of macular hole and vitreoretinal surgery was 30.3 ± 6.8 SD months (median 1.5 months, ranged from 1 to 144 months) in eyes with TMH and 2.6 ± 2.3 SD months (median 1 month, ranged from 1 to 6 months) in eyes with IMH.

The BCVA of patients with TMH was 0.8 ± 0.3 SD LogMAR preoperatively (median 0.7, ranged from 1.2 to 0.6 LogMAR) and 0.7 ± 0.4 SD LogMAR at last follow-up examination (median 0.6, ranged from 1.2 to 0.2 LogMAR, period of follow-up 6.6 ± 6.8 SD months, median 3 months, ranged from 1 to 17 months). Patients with IMH showed a preoperative BCVA of 0.9 ± 0.1 SD LogMAR (median 0.8, ranged from 1.0 to 0.7 LogMAR) and a BCVA at last follow-up examination of 0.8 ± 0.7 SD LogMAR (median 0.5, ranged from 2.0 to 0.3 LogMAR, period of follow-up 4.6 ± 3.4 SD months, median 6.0 months, ranged from 0 to 8 months). One patient was lost to follow-up and had a BCVA of 2.0 LogMAR 2 days after surgery with 90% of the vitreous cavity filled with C_2_F_6_. TMH showed a primary closure rate of 60% and IMH 80%, respectively. Patient 2 required a second vitrectomy in order to close the TMH. Table 1 shows postoperative BCVA of patient 2 after first vitrectomy. A correlation between cell count on ILM and macular hole closure rate was not determined.

### Immunocytochemistry

The immunocytochemical analysis as well as the cell count and area of each specimen are shown in Table 2.

In summary, eyes with TMH showed a mean cell count of 39.2 ± 49 SD (median 14 cells, ranged from 0 to 115 cells). Eyes with IMH presented with a mean cell count of 164.4 ± 186.4 SD (median 43 cells, ranged from 34 to 454 cells). The mean surface area in eyes with TMH was 3.4 ± 2.3 SD mm^2^ (median 3.7 mm^2^, ranged from 1.2 to 6.6 mm^2^) and in eyes with IMH 1.4 ± 0.7 SD mm^2^ (median 1.6 mm^2^, ranged from 0.3 to 2.2 mm^2^).

In the majority of eyes with TMH (3 of 5 eyes), positive immunoreactivity for the microglial cell marker anti-IBA 1 was seen. For macroglial cells, anti-GS was documented positive in 2 of 5 eyes and anti-VIM in 1 of 5 eyes. All eyes with IMH (5 of 5 eyes) revealed a positive immunoreactivity for anti-IBA 1. For anti-GS, 5 of 5 eyes and, for anti-VIM, 4 of 5 eyes with IMH showed a positive immunostaining. Negative controls revealed no immunoreactivity. Figure 1 presents the pre- and postoperative OCT scans (Fig. 1a–d) of patient no. 3 with TMH in combination with phase contrast (Fig. 1e) and positive immunoreactivity for the different markers (Fig. 1f–h). Figure 2 demonstrates the same of patient no. 9 with IMH.

Colocalisations were seen in both entities and are listed in Table 2. Traumatic macular hole specimens showed colocalisations of anti-IBA 1 and anti-VIM (Nos. 2, 3), of anti-GS and anti-VIM and anti-IBA 1 (No. 3) and of anti-GS and anti-IBA 1 (No. 5). Specimens of IMH presented colocalisations of anti-GS and anti-VIM (Nos. 7, 9) and of anti-GS and anti-IBA 1 (No. 10).

### Transmission electron microscopy (TEM)

Ultrastructure was analysed in all 10 specimens using TEM. Table 2 presents the results in detail.

**Fig. 3:**
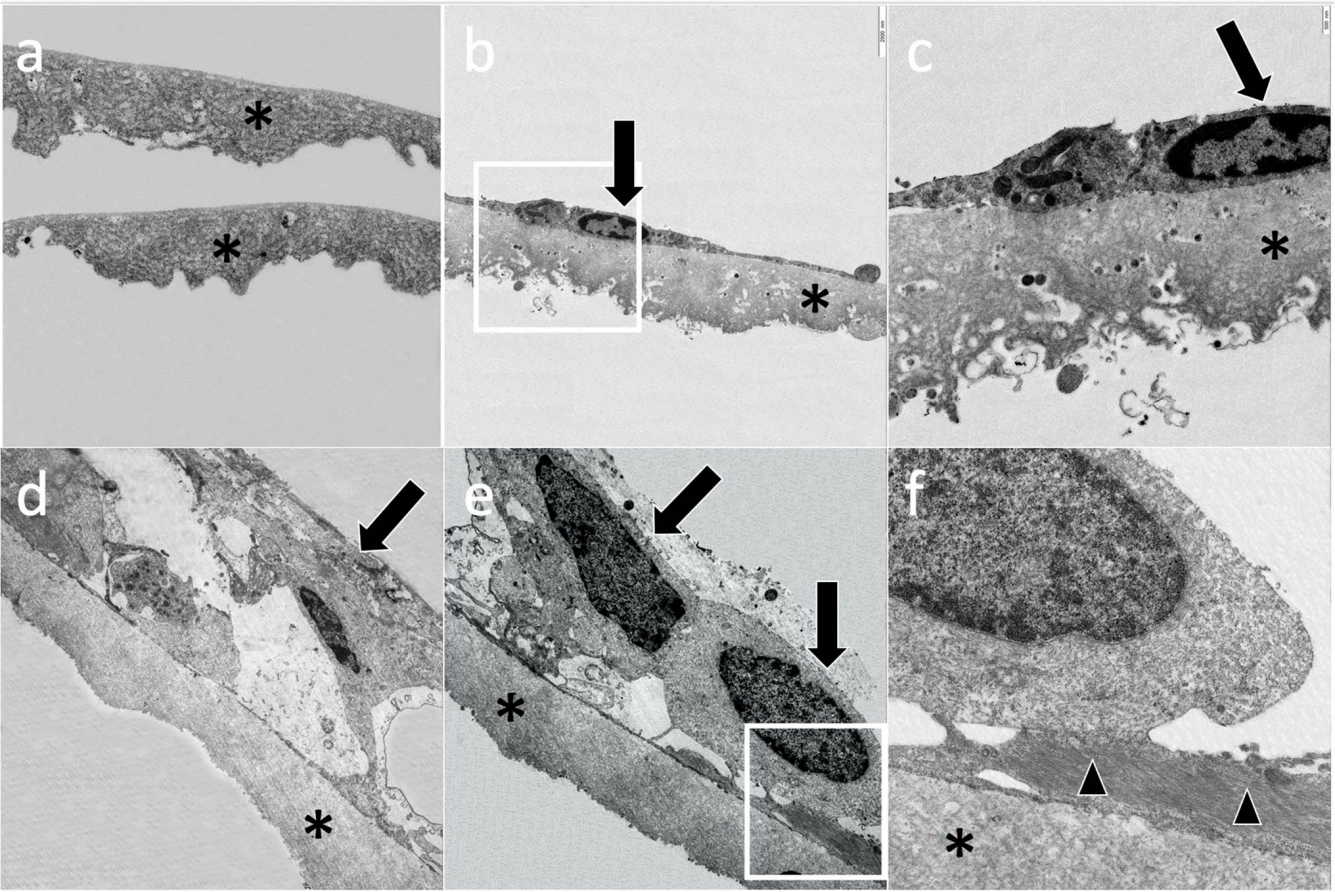
Transmission electron microscopy of specimens removed from **a**–**c** eyes with traumatic macular hole (TMH) and **d**–**f** eyes with idiopathic macular hole (IMH). **a** Internal limiting membrane (ILM) characterised by its undulated retinal side and its smooth vitreal side (asterisk). **b** Hyalocyte (arrow) situated on the vitreal side of the ILM (asterisk). **c** Magnification of the hyalocyte (arrow) with its cell nucleus and ILM (asterisk). **d** Cell multilayer (arrow) containing glial cells directly on the vitreal side of the ILM (asterisk). **e** Macroglial cells (black arrows) on the vitreal side of the ILM (asterisk). A cell extension of an astrocyte is shown between the hyalocyte and the ILM. **(f)** Magnification of hyalocyte with its cell nucleus, astrocyte (arrowheads) and ILM (asterisk). Original magnifications: **a**, **b**, **d**, **e** × 4400; **c**, **f** × 12,000

The ILM was found in all analysed specimens (Fig. 3) and was characterised by a smooth vitreal side and an undulated retinal side. There was no difference between the ILM of eyes with TMH and IMH. All detected cells were found on the vitreal side of the ILM. Neither cell debris nor cell bodies were seen on the retinal side of the ILM in both TMH and IMH.

In 3 of 5 specimens with TMH, single-layered glial cells were found at the VMI by series sections of the TEM (Fig. 3a). An exemplary hyalocyte on the vitreal side of the ILM of specimen no. 5 is shown in Fig. 3b and c. Hyalocytes were identified through a lobular cell nucleus, cytoplasmatic vacuoles, vesicles and mitochondria. Additionally, hyalocytes were recognised by cell runners, a distinct Golgi apparatus and a large amount of smooth and rough endoplasmatic reticulum. In the extracellular matrix, neither native vitreous collagen (NVC) nor thick collagen strands (CS) nor newly formed collagen (NFC) nor fibrous long spacing collagen (FLSC) was seen in eyes with TMH. In summary, 3 of 5 eyes with TMH showed ILM and cell single layers and no extracellular matrix in TEM. Specimens No. 1 and No. 4 showed just ILM and neither cells nor vitreous collagen in TEM, respectively.

In contrast, specimens of IMH showed multi-layered cells as well as cell clusters on the vitreal side of the ILM as illustrated in Fig. 3d–f. These cells were identified as astrocytes, hyalocytes and myofibroblasts (Fig. 3d–f). Myofibroblasts were characterised by rough endoplasmic reticulum, fusiform cell nucleus, aggregates of 5–7-nm subplasmalemmal cytoplasmic filaments with fusiform densities, and an absence of intracytoplasmic intermediate-type 10-nm filaments. In 1 of 5 specimens with IMH, only ILM without cells and extracellular matrix was detected (No. 6, Table 2). Extracellular matrix consisting of NVC was documented in 2 of 5 specimens (40%). NVC was characterised by regular arranged collagen fibrils with a diameter of less than 16 nm. Other collagen types such as NFC, CS or FLSC were not seen.

## Discussion

This clinicopathological study presents different immunocytochemical and ultrastructural findings at the VMI analysing surgically excised premacular tissue of ILM and ERM in eyes with TMH and IMH. Compared to a higher cell count and multi-layered cell composition in eyes with IMH, the fibrocellular composition shown in eyes with TMH revealed few single cells without tractive properties.

By immunocytochemistry, both entities presented a positive immunoreactivity for the macroglial cell markers anti-VIM and anti-GS as well as for the microglial cell marker anti-IBA 1. However, in eyes with TMH, a large variation of the cellular elements was seen. In general, a positive immunoreactivity of the used glial cell markers was found more often in eyes with IMH. In accordance, transmission electron microscopy (TEM) showed significantly more cellular structures identified as hyalocytes, astrocytes or myofibroblasts in specimens of IMH than of TMH.

To our knowledge, there is only one case series investigating the ILM in various maculopathies, among others an eye with TMH [21]. Kanavi and colleagues showed a positive immunostaining for the glial cell markers: anti-glial fibrillary acidic protein (anti-GFAP) and anti-neuron specific enolase (anti-NSE) [21]. Immunoreactivity for the myofibroblast marker anti-alpha smooth muscle actin (anti-α-SMA) and macrophage marker anti-CD68 was tested negative in this study [21].

Macroglial cells such as Mueller cells and astrocytes are neuronal cells and of a neuroectodermal embryonic origin. In contrast, microglial cells are non-neuronal cells with a mesodermal origin. They originate from macrophages that invade the brain during early development and have macrophage activity [30]. Microglia are known to reside in the inner and outer plexiform layers of the mature retina and play a neurotrophic role with maintaining the retinal homeostasis [31]. In the case of retinal stress, e.g. caused by macular hole development, the microglial cells get activated, proliferate and migrate to the place of action. At the same time, they release pro-inflammatory cytokines and are able to activate the aberrant complement pathway [31–35]. Whether activated retinal microglia are neurotrophic, neurotoxic or maybe both is still under discussion [31, 32, 35]. Previous studies determined the presence of macroglia and their neurotrophic potential in eyes with IMH [27–29, 36–38]. To our knowledge, there is no study about microglia markers in eyes with TMH.

Macroglia is identified by immunocytochemistry using several markers such as anti-GFAP, anti-VIM and anti-GS [38–40]. Anti-vimentin not only is specific for astrocytes like anti-GFAP, but also stains other macroglial cells such as Mueller cells [39]. Besides, it is known that activated microglia additionally express the filament vimentin [41, 42]. Glutamine synthetase is a Mueller cell-specific enzyme in the retina, found almost exclusively in Mueller glial cells [40]. In the literature, anti-IBA 1 is described as a specific marker for pan-macrophages and shows positive immunoreactivity with resting as well as activated microglia [43–45]. The IBA 1 protein is not found in neurons, astrocytes, Mueller cells or oligodendroglia [45–47].

In our study, all three analysed primary antibodies were tested positive in eyes with TMH and IMH. One specimen of TMH revealed no cells, so that immunocytochemistry could not be evaluated (No. 4). Another specimen of TMH only showed 5 cells, which were not detected with positive immunoreaction (No. 1). In IMH, positive immunoreactivity was seen more frequently in all specimens. These findings might allow the conclusion that macroglial and microglial cells are involved in the clinical course after TMH and IMH development. This is supported by the findings of colocalisations. Colocalisation of anti-VIM and anti-IBA 1 was found in 2 of 5 specimens of TMH, which might give evidence of the presence of activated microglia. In specimens with IMH, no colocalisation of anti-VIM and anti-IBA 1 was seen. The colocalisation of anti-VIM and anti-GS that was detected in some specimens of IMH and TMH might indicate the presence of Mueller cells. Unexpectedly, both entities showed the colocalisation of anti-GS and anti-IBA 1. Whether this colocalisation indicates the potential of microglia to endogenously express GS or demonstrates microglia that have previously phagocytised GS-positive cell debris still remains unclear. Though, microglia is not yet known to express the macroglia marker anti-GS.

In the literature, there is only one other known TEM case series that describes the analysis of ultrastructural details of the VMI of eyes with TMH, namely by Kumar and colleagues [22]. They identified ILM in all three examined specimens of eyes with TMH [22]. Similar to our ultrastructural results in which few single cells in only 3 of 5 specimens were found, they did not find cells nor ERM in any of the specimens [22]. Furthermore, they described NVC in 1 of 3 eyes with TMH [22]. However, our study revealed cells in the majority of specimens using the DAPI cell count analysis technique. The variance in our immunocytochemical and ultrastructural results could be explained by the technique of ultrathin series sectioning in which single cells in large flat-mounted specimens cannot be identified using TEM. In contrast, specimens of eyes with IMH presented higher cell counts (mean of 164 cells vs. 14 cells) and showed mono- and multi-layered cell composition consisting of myofibroblasts and hyalocytes, which is known from previous studies [28].

Furthermore, clinical data of the patients in our study revealed that the vitreous was attached to the posterior pole in the majority of eyes (60%) with TMH. However, it remains unclear whether PVD was induced by the ocular trauma or had already existed beforehand as the 2 patients (40%, Nos. 4 and 5) with preoperative PVD were patients older than 65 years. A high rate of an attached posterior vitreous body is in concordance with findings of previous studies. Huang and colleagues noted that the vitreous was still attached to the fovea in all included patients with TMH [9]. Yanagiya and colleagues found a foveal attached vitreous in 95% of patients with TMH [23] and Johnson and colleagues demonstrated a rate of vitreous attachment of 84% [8]. Thereby, it is assumed that an attached vitreous is an important marker for possible spontaneous closure, which should be considered in eyes with TMH. The results of our study support the hypothesis that an attached vitreous might serve as a bridge between the macular hole edges and thereby facilitates glial cell migration and proliferation [11]. This might also explain why patients with TMH have a high rate of spontaneous macular hole closure.

According to current data, there is no established therapy for TMH. In some cases, surgery might be needed, and in others, the TMH closes spontaneously. In fact, TMH do have a relatively high rate of spontaneous closure up to 50% within the first 9 months after development [7, 12–14, 20, 48]. In contrast, spontaneous closure of IMH appears in only 2.7–6.2% [11, 20, 49, 50]. Young age, a small diameter of the hole, little intraretinal fluid, no ERM and the absence of PVD, as mentioned above, seem to be common characteristics in patients presenting spontaneous closure of TMH [7, 11, 15–20]. But even in older patients and documented PVD, a spontaneous TMH closure is described in some case series [51–53]. This seems to be less probable than in children.

On the other hand, it is known that TMH persisting for more than 1 year are less likely to close spontaneously [14]. The recommended time of surgery varies between within the first 3 months and after an observational period of 6 months [7, 9, 14, 54, 55].

If surgery is needed, vitrectomy with fluid-gas exchange, gas tamponade and postoperative prone positioning, with or without ILM peeling or ILM flap, with adjunctive agents like TGFβ2 or autologous platelet concentrate and TMH closure using amnion-patches are all methods that showed positive results concerning TMH closure and BCVA gains in several studies [8, 56–63].

Limitations of our study include its relatively small number of analysed specimens, its retrospective analysis of clinical data and the variable period of follow-up. Furthermore, the collection of premacular tissue during pars plana vitrectomy might have been incomplete and could have led to incorrect numbers of cell count measurements. Additionally, it must be noted that due to the preparation of flat-mounted membranes, we were only able to test 3 different primary antibodies and decided to focus on glial cell markers. Comparing the cell count found via immunocytochemistry and TEM, it is likely that not every cell was cut through ultrathin serious sections of 60 nm for TEM. Therefore, we did not focus on statistical analysis and correlation of ultrastructural findings and functional parameters.

In conclusion, our results present less cells on the vitreal side of the ILM in eyes with TMH compared to multi-layered cell composition in eyes with IMH. Furthermore, we found a positive immunoreactivity for macro- and microglial cell markers in both entities. Clinical data revealed an attached vitreous body in the majority of eyes with TMH. Vitreous collagen was only detected in specimens of IMH but not in specimens of TMH, suggesting that an incomplete posterior vitreous detachment with vitreoschisis plays no role in the development of TMH.

## Data Availability

Not applicable.
